# AMEND: active module identification using experimental data and network diffusion

**DOI:** 10.1186/s12859-023-05376-z

**Published:** 2023-07-06

**Authors:** Samuel S. Boyd, Chad Slawson, Jeffrey A. Thompson

**Affiliations:** 1 Department of Biostatistics and Data Science, University of Kansas Medical Center, 3901 Rainbow Blvd., Kansas City, KS 66103 USA; 2 Department of Biochemistry, University of Kansas Medical Center, 3901 Rainbow Blvd., Kansas City, KS 66103 USA; 3 University of Kansas Cancer Center, Kansas City, KS USA; 4 University of Kansas Alzheimer’s Disease Research Center, Fairway, KS USA

**Keywords:** Network analysis, Module identification, Omics

## Abstract

**Background:**

Molecular interaction networks have become an important tool in providing context to the results of various omics experiments. For example, by integrating transcriptomic data and protein–protein interaction (PPI) networks, one can better understand how the altered expression of several genes are related with one another. The challenge then becomes how to determine, in the context of the interaction network, the subset(s) of genes that best captures the main mechanisms underlying the experimental conditions. Different algorithms have been developed to address this challenge, each with specific biological questions in mind. One emerging area of interest is to determine which genes are equivalently or inversely changed between different experiments. The equivalent change index (ECI) is a recently proposed metric that measures the extent to which a gene is equivalently or inversely regulated between two experiments. The goal of this work is to develop an algorithm that makes use of the ECI and powerful network analysis techniques to identify a connected subset of genes that are highly relevant to the experimental conditions.

**Results:**

To address the above goal, we developed a method called Active Module identification using Experimental data and Network Diffusion (AMEND). The AMEND algorithm is designed to find a subset of connected genes in a PPI network that have large experimental values. It makes use of random walk with restart to create gene weights, and a heuristic solution to the Maximum-weight Connected Subgraph problem using these weights. This is performed iteratively until an optimal subnetwork (i.e., active module) is found. AMEND was compared to two current methods, NetCore and DOMINO, using two gene expression datasets.

**Conclusion:**

The AMEND algorithm is an effective, fast, and easy-to-use method for identifying network-based active modules. It returned connected subnetworks with the largest median ECI by magnitude, capturing distinct but related functional groups of genes. Code is freely available at https://github.com/samboyd0/AMEND.

**Supplementary Information:**

The online version contains supplementary material available at 10.1186/s12859-023-05376-z.

## Introduction

High-throughput technologies continue to produce vast quantities of molecular data, be it genomic, transcriptomic, proteomic, or otherwise. These different omics data help to reveal a complex, interconnected cellular landscape, and the analysis of omics data can highlight specific molecular features that may be implicated in a disease or biological condition [1–3]. In parallel with the proliferation of omics data, there exist large and expanding databases containing protein–protein interactions (PPI) [4–6]. These PPIs are determined by incorporating several different evidence types (e.g., high-throughput experiments, co-expression analysis, database imports) and are often represented in the form of graphs (i.e., networks), which are mathematical objects consisting of nodes (representing proteins) and edges (representing interactions) connecting them [7]. However, network analysis involving only PPI networks is limited when the goal is to study a particular biological process, since they are static representations of interactions within a cell and thus cannot elucidate the molecular features involved in a specific biological context. For example, topological clustering algorithms on PPI networks are not expected to capture sets of proteins showing differential abundances between tumor and normal groups. The integration of gene expression data and PPI networks has emerged as a powerful way to overcome this limitation by boosting signal in experimental data and making network analysis results context-specific. This is most often done by attributing gene-wise summaries of the experimental data (e.g., log2 fold change) as weights to the nodes in the network and finding subsets of connected nodes with relatively large weights. Methods falling within this analysis paradigm have been termed active module identification (AMI) methods, with *active* referring to molecular features relevant to the biological process being studied, and *module* referring to the subset of connected nodes obtained from the analysis [8].

A powerful analytic framework used within AMI methods is network diffusion (also called network propagation). It is based on the assumption of ‘guilt by association,’ in which molecular features (e.g., proteins) are assumed to have functional similarities with their direct interacting partners in the network [9, 10]. This concept can be extended beyond direct interactions to consider the network as a whole. In this framework, node-wise experimental values are diffused through the edges to other nodes in the network, with node weights after diffusion representing their affinity, or closeness, to other highly weighted nodes. This method of re-weighting nodes through diffusion takes into account prior information, in the form of experimental data (e.g., omics data), and topological information, since node weights must propagate through the edges. A popular network diffusion approach is random walk with restart (RWR), which allows for control over the extent to which experimental values are spread to other nodes.

There currently exists an abundance of AMI methods that have been developed on various data types using different frameworks to filter out genes. jActiveModules was developed for microarray data and heuristically finds high scoring subnetworks through simulated annealing [8]. These subnetworks are scored by computing an average z-score of the p-values coming from the microarray experiment. BioNet fits a beta-uniform mixture model using *p* values from a differential expression (DE) analysis to score each node in the network and then uses integer linear programming to optimally solve the Maximum-weight Connected Subgraph (MWCS) problem [11]. The MWCS problem aims to identify a subset of nodes of maximum node weight that are connected, where connected means there exists a path between any two nodes in the subgraph. HotNet2 was developed for somatic mutation data and implements RWR on the interaction network, from which a weighted directed graph is created [12]. Strongly connected components of this graph are then identified and assessed for statistical significance. NetCore also implements RWR and obtains permutation-based p-values for the propagation scores, from which a set of modules is derived by thresholding these *p* values and scores [13]. DOMINO takes as input a list of differentially expressed genes and applies various clustering and network diffusion algorithms to arrive at a set of final modules [14].

Despite the advantages of combining gene expression and PPI data, there can be drawbacks associated with AMI methods. NetCore and HotNet2, among others, apply a threshold to propagation results in order to select a subset of genes [12, 13, 15]. These thresholds can be arbitrary, and the results may be sensitive to the choice of threshold value. Furthermore, the best threshold value is context dependent. Also, AMI methods using network diffusion often run the diffusion process only once on the full PPI network, which contains many proteins and interactions not relevant to the biological condition being studied. This can introduce noise into the network analysis. Finally, DOMINO binarizes the experimental data based on the results of a DE analysis, which leads to information loss [14].

AMI methods are often developed and evaluated using a specific data type (e.g., mutation data for HotNet2, gene expression data for jActiveModules) or metric (e.g., *p* values, log2 fold change), allowing them to answer specific biological questions. A recently proposed continuous metric, the equivalent change index (ECI), computes ratios of log2 fold changes in order to compare effect sizes between experiments, allowing researchers to determine the degree to which genes are equivalently or inversely changed [16]. This can be used to validate results from a previous study or to compare how two treatments affect gene expression. No AMI method has been designed for use with the ECI, which serves in part as motivation for this study. In the context of AMI, the ECI will allow us to determine active modules that are similarly or inversely regulated by different treatments (for example, it would allow us to find which modules are affected by two similar drugs or modules that are upregulated in a disease but downregulated by a drug).

To address the unmet need of AMI methods that are designed for use with the ECI, we introduce AMEND, an algorithm that utilizes the ECI to identify active subnetworks of genes perturbed in similar or opposing ways across two experiments. AMEND does not rely on arbitrary thresholding but rather iteratively performs network diffusion for gene selection. To evaluate our proposed method, we benchmarked its performance against NetCore and DOMINO using two biological datasets: a GLUT4 knockout-overexpression microarray dataset and an RNA-seq dataset investigating antidepressants.

## Methods

This section will begin with a data description, followed by an explanation of the various components of the AMEND algorithm, and ending with descriptions of the benchmark and evaluation methods.

AMI methods require two types of data as input: experimental data and a molecular interaction network. For the purposes of this study, we will focus on microarray/RNA-seq gene expression data and PPI data. Specifically, we will extract a gene-wise summary of the gene expression data that compares effect sizes between two experiments, called the Equivalent Change Index (ECI).

### Equivalent change index

The ECI is a gene-wise measure of equivalent or inverse change in expression levels between two experiments [16]. It ranges between − 1 and 1, with a value of − 1 indicating changes in expression in exactly opposing ways (e.g., expression was halved between groups for one experiment but doubled for the other), and a value of 1 indicating changes in expression in exactly equivalent ways (e.g., expression was doubled between groups for both experiments).

Let $\beta_{ij}$ and $p_{ij}$ be the log_2_ fold change and the *p* value, respectively, for gene $i$ from experiment $j$. Then the ECI for the *i*th gene is

$$\lambda_{i} = sign\left( {\beta_{i1} \times \beta_{i2} } \right) \times \frac{{\min \left( {\left| {\beta_{i1} } \right|,\left| {\beta_{i2} } \right|} \right)}}{{\max \left( {\left| {\beta_{i1} } \right|,\left| {\beta_{i2} } \right|} \right)}} \times \left( {1 - \max \left( {p_{i1} ,p_{i2} } \right)} \right)$$

Since the ECI is simply the weighted ratio of effect sizes between two experiments, this can accommodate data from different levels of cellular description (e.g., transcriptome, proteome, metabolome). It can be particularly suitable for knockout (KO)/over-expression (OX) experiments, where one clearly expects inverse or equivalent change between the KO versus control and OX versus control groups. The ECI applied to gene expression data will be the main experimental input for the AMI methods used in this study, although they are generalizable to other data types. ECI significance testing can be performed using a bootstrap approach [17]. In this study, ECI values were derived from the following two data sets.

### Glucose transporter-4 (GLUT4) data

The GLUT4 microarray gene expression data was obtained from the National Center for Biotechnology Information-Gene Expression Omnibus (NCBI-GEO) database (AC NO: GSE35378) and derives from an adipose tissue GLUT4 KO-OX experiment in mice by Herman et al. [18–20]. GLUT4 is activated by insulin and serves to bring glucose into the cell. Alterations to GLUT4 expression levels are associated with insulin sensitivity [21]. These data were produced with the expectation of opposing changes between the two experimental conditions. Differential expression analysis for each treatment versus control was performed using the *limma* package in R, whereby log_2_ fold changes were obtained [22]. These log_2_ fold changes were then used to calculate the ECI.

### Anti-depressant data

The second data set focuses on the effects of two anti-depressants, ketamine and imipramine, on gene expression for several brain regions in mice subjected to chronic social defeat stress. The analysis was limited to prefrontal cortex samples, as these were the most numerous. The data were produced by Bagot et al. [23] using the Illumina HiSeq 2500 platform and are available from the NCBI-GEO database (AC NO: GSE81672). With the two drugs both being anti-depressants, some equivalent changes are expected in the data. Differential expression analysis was conducted using the *edgeR* package in R [24–26].

These two datasets were chosen to highlight two possible use-cases of the ECI: knockout-overexpression experiments and the comparison of two drugs. Moreover, these datasets are amenable to use with the ECI since each includes two treatment–control arms.

### STRING PPI networks

In this study, we used the STRING database (v11.0b) for the construction of high-confidence PPI networks [6]. STRING is a freely available resource that contains PPIs for thousands of organisms. While there are many similar databases available, STRING has been shown to be a top performer in terms of recovering literature-curated disease gene sets [27]. PPIs are annotated with confidence scores derived from topological, experimental, and annotation-based sources [7]. For this study, only interactions with a combined confidence score $\ge$ 0.8 were kept, in an effort to create a high-confidence PPI network.

To construct the PPI networks, only proteins mapping to genes in the experiment were included, since AMEND requires that each protein have a gene-wise summary from the experimental data. Therefore, there are two separate PPI networks used in this analysis, corresponding to the GLUT4 and Anti-depressant datasets. Each of the initial networks was disconnected, meaning there were two or more subnetworks (i.e., components) within the whole network with no edges connecting them. AMEND requires a connected network, so only the largest connected component was kept for each network. For the GLUT4 data, this resulted in a network of 6381 proteins and 118,657 interactions. The PPI network for the Anti-depressant data included 10,152 proteins and 115,206 interactions.

The ECI values from each dataset are assigned to the nodes of their respective PPI networks as node weights. These networks are the unified representations of the transcriptomic and PPI data. However, they do not indicate the set of interactions that are most relevant and active in a specific biological context. This is where the AMEND algorithm comes in.

### Module identification with AMEND

**Fig. 1:**
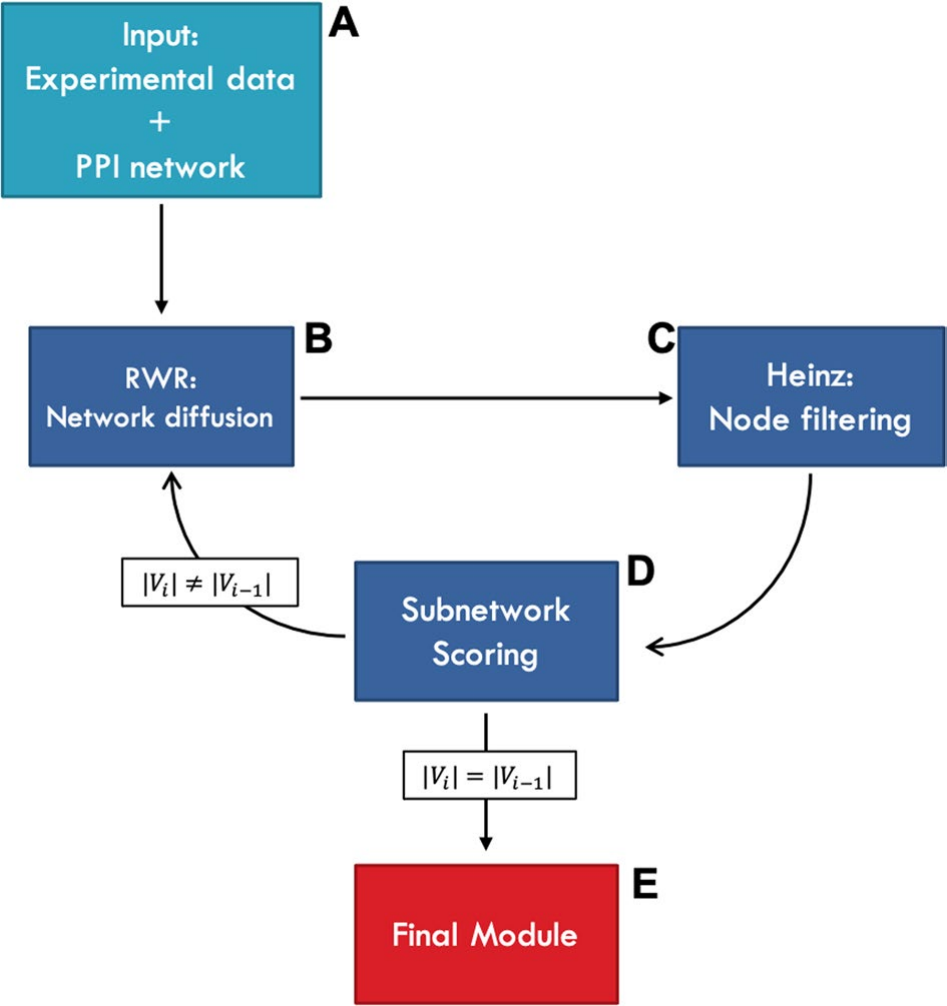
AMEND workflow. **A** Map gene-wise summaries of experimental data (e.g., ECI, log_2_ fold change) to proteins in the PPI network. At this stage, compute standardized ECI values for use in module scoring. **B** Perform RWR. First, transform untreated ECI values into seed values, which must be non-negative and sum to 1. Second, for a given restart value, run RWR and get node weights. Third, shift the weights down by a certain quantile determined by an exponential decay schedule, guaranteeing some positively and some negatively weighted nodes. The restart value is determined by running steps B–D over a grid of values, choosing the one resulting in the largest subnetwork score. **C** Run Heinz, a heuristic solution for finding a maximum-weight connected subnetwork. Use the shifted node weights obtained from step B. This will return a connected subnetwork. **D** Score the subnetwork. The score of a network is the product of the mean standardized ECI (calculated in step A) and the mean core-clustering coefficient, a node-wise measure of the degree to which the neighbors of a node are connected to one another. If there is a change in network size ($|{V}_{i}|$ is the size of subnetwork $i$), continue to RWR for the next iteration, or else break out of the loop and go to step E. **E** The final module is chosen among the subnetworks generated at each iteration by taking the one with the largest score

AMEND makes use of two previously existing network analysis methods in an iterative manner: Random walk with restart (RWR) [28, 29], and *Heinz* (heaviest induced subgraph) [11]. These are the main mechanisms that will select which genes are included in the final subnetwork. RWR calculates node weights based on experimental and topological information, while *Heinz* attempts to find the maximum-weight connected subgraph using the node weights derived from RWR. The resulting subnetwork is scored and input into RWR for the next iteration. The process stops when there is no change in network score between successive iterations, and the highest-scoring network is returned as the final module. RWR and *Heinz* will be described subsequently, followed by a description of network scoring. Figure 1 provides a diagram outlining the workflow of AMEND.

### Random walk with restart

RWR takes as input a PPI network in the form of an adjacency matrix (weighted or unweighted) and a vector of seed weights. It can be classified under the category of network diffusion methods, wherein node weights are diffused to other nodes by way of the network topology. RWR simulates random walkers starting from a set of seed nodes with probability given by the seed weights. At each step, the walkers can move to a neighboring node with a certain probability (determined by the network topology), or it can return to the seed nodes with probability $\alpha$: the restart probability. The steady-state probability vector can be obtained by iterative matrix multiplication and represents each node’s affinity to the seed nodes. At step $i$, the affinity vector is denoted by

$$P_{i} = \left( {1 - \alpha } \right)A_{N} P_{i - 1} + \alpha P_{0}$$

where $A_{N}$ represents a column-normalized transition matrix, $P_{i}$ the affinity vector at step $i$, $P_{0}$ the vector of seed values, and $\alpha$ the restart probability. The steady-state affinity vector $P_{i}$ is reached when $\left| {P_{i} - P_{i - 1} } \right| \le \epsilon$ for some $\epsilon > 0$. The elements of $P_{i}$ are called propagation scores, representing the re-weighting of nodes after network propagation. A key characteristic of AMEND is that it allows $\alpha$ to vary at each iteration, by way of a grid search (see *Setting the restart parameter*).

An important step in RWR is the normalization of the adjacency matrix to create a transition matrix. A common choice is degree normalization, where each column of the adjacency matrix is divided by the sum of the elements in that column. However, protein–protein interactions have been shown to exhibit technical and ascertainment biases, which can lead to degree bias in PPI networks [30]. The AMEND algorithm uses the normalization scheme recommended by Barel et al. [31], which utilizes node coreness, a global measure of how centrally located a node is in the network. They showed that core normalization outperforms degree normalization in terms of identifying GWAS gene sets [13]. Coreness and degree are positively correlated, but a node can have a high degree and low coreness (e.g., star-like graphs). In order to combat degree bias, a core-normalization scheme was implemented. For each column of the adjacency matrix, the non-zero elements are replaced by the coreness of the nodes associated with those rows. The column is then divided by the sum of its new elements. Mathematically, this core-normalized matrix is represented by

$$\left( {A_{N} } \right)_{ij} = \frac{{k_{i} }}{{\mathop \sum \nolimits_{{l;A_{lj} \ne 0}}^{{}} k_{l} }}$$

where $k_{i}$ denotes the coreness of node $i$, and $A_{lj}$ denotes the $lj$th element of the original adjacency matrix. The $ij$th element of the core-normalized adjacency matrix represents the probability of a random walker moving from node $j$ to node $i$.

Another important consideration is the choice of values for the seed vector. The seed vector is always normalized to sum to one, so the only requirement is that the values be non-negative, with at least one non-zero element. The choice of seeding scheme will depend on the biological question being addressed and the nature of the data. For example, in the context of ECI, one may be interested in inverse change. Thus, values closer to − 1 should be given more weight than those closer to 1. Simply shifting and scaling all values uniformly would give more weight to ECIs of 0 than to ECIs not in the direction of interest (DOI). However, we are assuming that ECIs not in the DOI are still more biologically relevant than ECIs of zero. Considering this, a possible seeding scheme is to take the absolute value of the ECIs, multiply the values that were not in the DOI (positive values in this example) by some constant factor between 0 and 1, thereby reducing their weight, and then scaling so that the vector sums to 1. More formally, the *i*th element of the seed vector $P_{0}$ can be given by

$$p_{oi} = \frac{{\left| {e_{i} } \right| \times I\left( {s \times e_{i} > 0} \right) + c \times \left| {e_{i} } \right| \times \left( {1 - I\left( {s \times e_{i} > 0} \right)} \right)}}{{\mathop \sum \nolimits_{j = 1}^{n} \left| {e_{j} } \right| \times I\left( {s \times e_{j} > 0} \right) + c \times \left| {e_{j} } \right| \times \left( {1 - I\left( {s \times e_{j} > 0} \right)} \right)}}$$

where $e_{i}$ is the ECI of gene $i$, $s$ is the sign of the DOI, $I\left( \cdot \right)$ is an indicator function, and $c \in \left[ {0,1} \right]$ is a constant representing the relative weight given to genes not in the DOI compared to those in the DOI. For example, when interested in negative ECIs ($s = - 1$), $c = 0.5$ translates into weighting a positive ECI gene half that of a negative ECI gene of equal magnitude. This is the seeding scheme used in this study, with $c = 0.5$.

After diffusion, the RWR scores are shifted by the *k*th quantile, where $k \in \left[ {0,1} \right]$. The shifted RWR scores are given by

$$p_{j}^{^{\prime}} = p_{j} - Q_{k} \left( P \right)$$

where $p_{j}$ is the *j*th element of the steady-state affinity vector $P$, and $Q_{k} \left( P \right)$ is the *k*th quantile of $P$. This is to ensure the presence of both positive and negative scoring nodes, which is a requirement for *Heinz*. The quantile *k* decreases at each iteration of AMEND, following an exponential decay schedule, and can be viewed as a filtering rate (see *Setting the filtering rate schedule*). As *k* decreases, fewer nodes have negative scores after RWR, resulting in a larger subnetwork from *Heinz*, which means fewer nodes are filtered out. The shifted RWR propagation scores $p_{j}^{^{\prime}}$ will serve as node weights in *Heinz*.

#### Heaviest induced subgraph (Heinz)

*Heinz* attempts to provide a solution for the MWCS problem. Dittrich et al*.* [11] describe an exact approach that transforms the MWCS problem into the Prize-collecting Steiner Tree (PCST) problem, a solution for which is provided by Ljubić et al*.* [32]. This exact approach is computationally intensive, however, and access to the necessary software may not be available. AMEND therefore adapts its solution to the MWCS problem from the heuristic approach given by [33]. It takes as input a graph with both positive and negative nodes and proceeds by collapsing connected positive nodes into single meta-nodes and finding minimum spanning trees of this transformed graph, with edge weights derived from the weights of the incident nodes. It returns a connected subgraph.

#### Network scoring

To evaluate the quality of a subnetwork, we introduce a network scoring function that considers both experimental and topological information, which are represented by the average standardized ECI and the average core-clustering coefficient, respectively. The ECI values are standardized with respect to the entire data set, and if necessary, they are multiplied by − 1 to ensure ECI values in the DOI have positive values. The core-clustering coefficient is a measure implemented in the highly utilized topological clustering algorithm MCODE [34]. It is the edge density of the largest *k*-core of the immediate neighborhood of a node. Unlike the clustering coefficient, the core-clustering coefficient of a densely-connected node is not reduced by the presence of sparsely connected neighbors. Formally, the network scoring function is given by

$$f\left( G \right) = \overline{Z}_{G} \times \overline{C}_{G}$$

where $G$ is the network to be scored, $\overline{Z}_{G}$ is the average standardized ECI of the nodes in $G$, and $\overline{C}_{G}$ is the average core-clustering coefficient of the nodes in $G$.

#### Setting the restart parameter

An important characteristic of AMEND is that the restart parameter for RWR is allowed to vary between iterations. This parameter controls how much the experimental weights are diffused throughout the network. The input network is changing at each iteration, so it is reasonable to assume that the optimal restart parameter value will change as well. A grid search is used to set this parameter. For each grid value, RWR is run, producing node weights for *Heinz*, which gives a subnetwork that is scored. The grid value resulting in the highest-scoring subnetwork is chosen.

#### Setting the filtering rate schedule

**Fig. 2:**
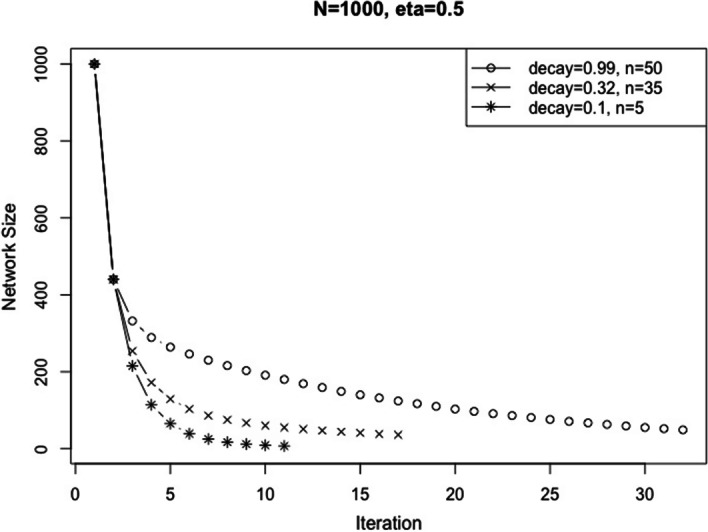
AMEND simulated behavior with different decay values. As the decay increases, the algorithm converges on larger module sizes, since a larger decay means the filtering rate drops off more drastically each iteration, which means fewer nodes are filtered out, giving a larger final module

The module returned by AMEND is sensitive to the sequence of quantiles (i.e., filtering rates) used to shift the RWR scores at each iteration. An exponential decay schedule is used to determine this sequence. Formally, the filtering rate at iteration *i* is given by

$$f\left( {i,\eta_{0} ,d} \right) = \eta_{0} \times e^{{ - d\left( {i - 1} \right)}}$$

where $\eta_{0}$ is the starting filtering rate and *d* is the decay parameter. Given $\eta_{0}$, we set the decay to be the maximum value that will allow the algorithm to arrive at a module of size *n.* To determine this maximum decay value, we note that the filtering rate represents the proportion of nodes with negative weights, and *Heinz* attempts to find a subnetwork that excludes as many negatively weighted nodes as possible. Therefore, we let the filtering rate be an approximation for the proportion of nodes removed at a given iteration. So, for a given $\eta_{0}$ and *d*, it is possible to simulate the sizes of the subnetworks at each iteration by iteratively multiplying the size of the current network by $1 - f\left( {i,\eta_{0} ,d} \right)$ and stop when there is no change in network size. Figure 2 shows the simulated behavior of the algorithm starting with a network of 1000 nodes and a filtering rate of 0.5. As *d* increases, the final module size increases, indicated by increasing horizontal asymptotes in Fig. 2. We increase *d* until the simulated final module size is greater than or equal to *n,* which is a parameter set by the user and a good approximation of the size of the observed final module.

The starting filtering rate is determined by particle swarm optimization (PSO), which is a computational method that attempts to optimize an objective function by iteratively updating candidate solutions, called particles, based on their current function value and the function values of the other particles [35]. In the context of AMEND, the objective function to maximize is the final module score, and the particles represent candidate starting filtering rates. PSO was used instead of a classic grid search out of efficiency considerations, since each run of AMEND is computationally intensive and PSO can search the search-space more thoroughly than a grid search in the same number of runs (see Additional files 1 and 2).

### Benchmark methods

AMEND was compared to other module identification methods that rely on network diffusion. The chosen methods were NetCore and DOMINO [13, 14]. NetCore takes experimental values as input and uses RWR (with core normalization) to propagate experimental values to other nodes in the network. The same seed scheme used for RWR in AMEND is used here. It then assigns an empirical p-value to the propagation scores by applying RWR on 100 random degree-preserving networks. Starting from the top 100 nodes based on their seed weights (called the seed subnetwork), new nodes are added if they share at least one connection with a node in the seed subnetwork, have propagation weights exceeding some threshold (defaults to the 75th percentile of the propagation weights of the significant nodes not already in the seed subnetwork), and have an empirical *p* value below 0.01. The connected components of this extended subnetwork are returned as modules.

DOMINO requires a list of “active” genes, and what is defined as active will depend on the biological question of interest. For this study, when we are interested in inverse change, only the significant, negative ECI genes are included in the input list. Similarly, when equivalent change is of interest, only significant, positive ECI genes are included in the input list. It first applies the Louvain clustering algorithm to the PPI network to obtain subsets of nodes called slices [36]. Slices are retained only if they contain a large enough proportion of active nodes. For the retained slices, a network diffusion approach is used that ‘activates’ neighbors of active genes based on a linear threshold model. The PCST problem is heuristically solved using diffusion results as node weights, with edge costs being 0 if the edge is incident to an active gene and 1 − $\epsilon$ otherwise, where $\epsilon > 0$. The resulting subgraphs from the PCST solutions are termed sub-slices. The Girvan-Newman clustering algorithm is then applied to these sub-slices that have greater than 10 nodes [37]. These sub-slices and clusters of sub-slices are then tested for overrepresentation of active genes using a hypergeometric test, adjusting for multiple testing. The significant sub-slices are returned as the final modules.

### Module evaluation

To evaluate the quality of a module in terms of experimental information, the median ECI and the proportion of significant ECI genes are used as evaluation metrics. These are equitable and relevant metrics since each method uses either the continuous ECI values or a list of significant ECI genes as input and the goal is to find genes with extreme ECI values. Additionally, a module’s relevance to known sets of functionally similar genes (pathways) is assessed with overrepresentation analysis (ORA), which considers the overlap between pathways and modules by way of a hypergeometric test. Pathways are obtained from Reactome [38]. All genes from the PPI network are included as background, and only pathways with an adjusted *p* value $\le 0.05$ are considered significant, using the Benjamini–Hochberg method for multiple testing [39].

ORA is a commonly used method to functionally characterize modules returned from AMI methods. However, it has been observed that there can be a high overlap between enriched pathways from the original data and from permuted data, which implies that ORA results may be spurious [14]. To assess the validity of ORA results, the authors of DOMINO proposed the empirical-to-hypergeometric ratio (EHR), which measures the proportion of pathways significantly enriched for the original data but not for permuted data. For a given dataset and AMI method, the original data is permuted, and modules are obtained. ORA is applied to each module, with a pathway’s overall enrichment score among the different modules being the maximum value of $- \log_{10} \left( {pval} \right)$. This is repeated on *M* randomly permuted datasets (*M* = 1000 for this study) to obtain a null distribution of enrichment scores for each pathway. Enrichment scores are also obtained from the original dataset. A pathway’s empirical *p* value is the proportion of enrichment scores from the empirical null distribution (obtained from permuted data) that are greater than the enrichment score from the original data. A pathway is called *empirically validated* (EV) if its empirical *p* value is less than or equal to 0.05 and if its adjusted *p* value (the minimum across all modules) from a hypergeometric test on original data is less than or equal to 0.05. The EHR is the proportion of the pathways significantly enriched for any of the original modules that are also EV pathways. While this gives an overall measure of the quality of the results returned by ORA, disconnected subnetworks will often be functionally characterized individually. The module-level EHR (mEHR) is the proportion of the pathways significantly enriched for a given module that are also EV pathways, with EV having the same definition as in EHR.

### Consistency analysis

A desired quality of AMI methods is consistency; given two or more independent datasets designed to study a similar biological condition, will the resulting modules be similar? Although the comparison of data captured in different labs from different specimens at different times is subject to batch effects, an AMI method can analyze these data in the shared context of a PPI network, which (it is hoped) will increase robustness to noise and give comparable results. To analyze the consistency of AMEND, we considered four independent gene expression datasets from NCBI GEO (GSE197016 [40], GSE140457 [41], GSE21636 [42], GSE147709 [43], henceforth designated as datasets 1–4, respectively), all from different platforms and all containing normal and BBN-treated mouse bladder samples (BBN is a chemical used to induce bladder tumors in mice). There were also differing dosages and mouse strains between the experiments. We measured the similarity of two modules obtained by using the ECIs calculated from two pairs of datasets (e.g., ECI from datasets 1 and 2, and ECI from datasets 3 and 4; 3 total combinations). This was done for various approximate final module sizes (N = 10,20,30). Modules being compared were derived from a common PPI network from STRING [6]. Two similarity measures were used. The Jaccard Index measures the similarity of sets, while the Nested Index measures the degree to which one set is nested within another. The Jaccard and Nested Indices are respectively given by.

$J\left( {A,B} \right) = \frac{{\left| {A \cap B} \right|}}{{\left| {A \cup B} \right|}}$; $NI\left( {A,B} \right) = \frac{{\left| {A \cap B} \right|}}{{{\text{min}}\left( {\left| A \right|,\left| B \right|} \right)}}$.where A and B are sets of nodes representing modules.

To assess the statistical significance of each index value, a bootstrap procedure was performed. In detail, each module can be represented as a binary vector of length *N* (the number of nodes in the PPI network) with an element being 1 if that node is in the module, 0 otherwise. For each module obtained from the data, *B* samples of size *N* are drawn with replacement from the elements of the associated binary vector (*B* = 100,000). Jaccard and Nested Indices are then computed on these bootstrapped samples. The *p* value for an index is the proportion of bootstrapped values that are greater than or equal to the original index value. Furthermore, we evaluated the biological relevance of the non-overlapping genes to assess the validity of the results. ORA was performed on all modules returned from the BBN data, excluding the shared genes, and we recorded the number of times each pathway was returned as statistically significant.

### AMEND implementation

AMEND was developed using the R programming language [44]. It is freely available at https://github.com/samboyd0/AMEND.

## Results

### GLUT4 data

**Fig. 3:**
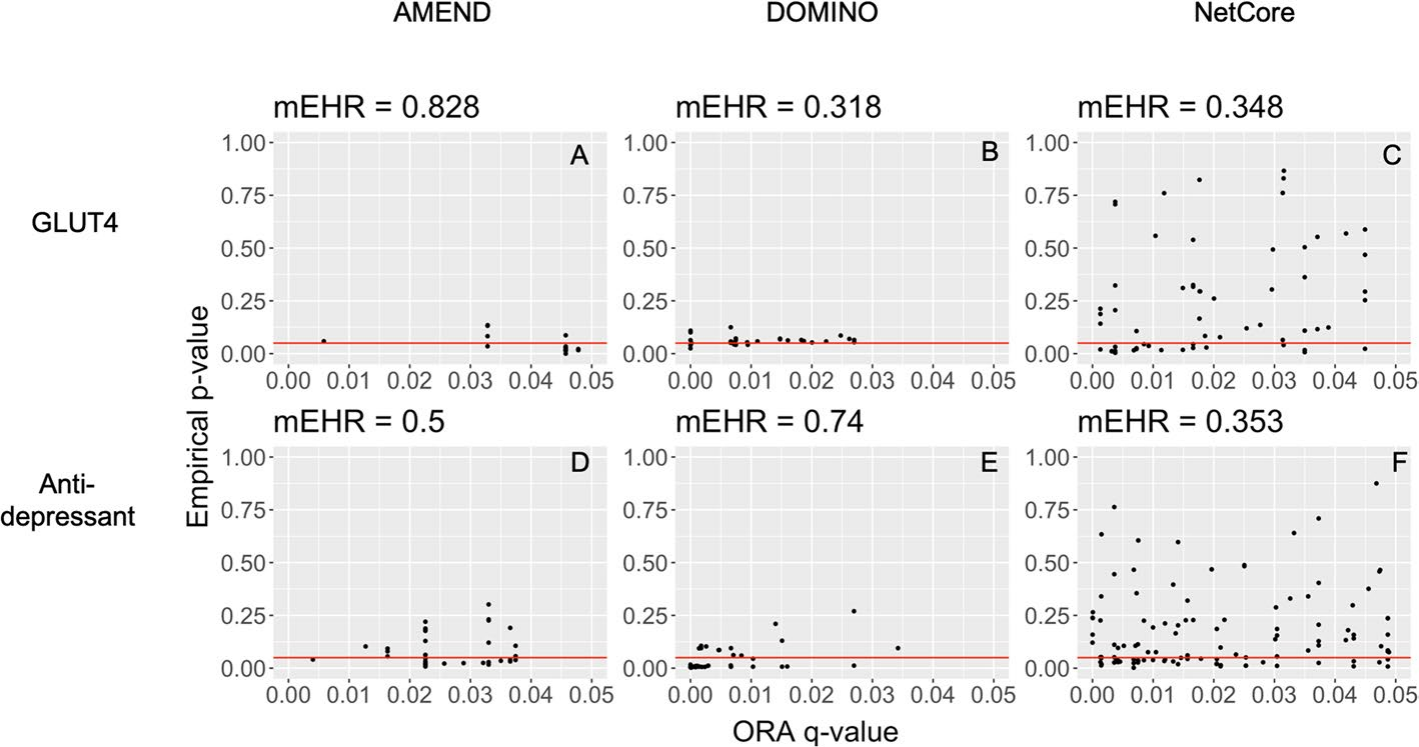
Module-level empirical-to-hypergeometric ratio (mEHR) for selected modules, where each point is a pathway returned from ORA. Pathways below the red line are EV pathways. **A** AMEND GLUT4, **B** DOMINO GLUT4 module 2, **C** NetCore GLUT4 module 1, **D** AMEND Antidepressant, **E** DOMINO Antidepressant module 1, **F** NetCore Antidepressant module 1

For the GLUT4 data, we are primarily interested in genes that were inversely changed between the GLUT4-KO vs. control and GLUT4-OX versus control groups. Thus, we are interested in genes with ECIs close to − 1. The PPI network for this data consists of 6381 proteins and 118,657 interactions. AMEND, NetCore, and DOMINO were applied to this network and returned either a single, connected module (AMEND), or several disconnected modules (NetCore, DOMINO). Table 1 contains some basic network statistics along with evaluation metrics. The parameter *n* (approximate final module size) was set to 15; therefore, it is not surprising that the AMEND module contains fewer nodes than those returned by the other methods. Smaller values of *n* tend to return smaller modules with median ECIs larger in magnitude, and the user may want to try several values to get a set of modules from which to choose. Overall, NetCore returned more highly negative ECI genes than DOMINO, while DOMINO returned a larger proportion of significant ECI genes than NetCore. AMEND was best able to capture both the high-magnitude and statistically significant ECI genes. Figure 3 shows empirical *p* values and ORA q-values for pathways enriched for select modules. Pathways associated with the AMEND and DOMINO modules tend to have lower empirical *p* values than those for NetCore.

**Table 1:** GLUT4 data module statistics

Module	Nodes	Edges	Module count	Edge density	Median ECI	Proportion significant	EHR/mEHR	Pathway count
AMEND, n = 15	15	23	1	0.219	− 0.715*	0.8*	0.828	29
Netcore, all	111	164	12	0.027	− 0.62	0.432	0.397	31
Netcore, m1	84	147	1	0.042	− 0.621	0.393	0.348	66
Netcore, m2	2	1	1	1	− 0.499	0.5	0	0
Netcore, m3	2	1	1	1	− 0.546	0.5	1*	2
Netcore, m4	2	1	1	1	− 0.515	0.5	0	0
Netcore, m5	2	1	1	1	− 0.686	1	0	0
Netcore, m6	3	2	1	0.667	− 0.671	0.667	0	2
Netcore, m7	5	5	1	0.5	− 0.613	0.6	1*	4
Netcore, m8	3	2	1	0.667	− 0.327	0.333	0	0
Netcore, m9	2	1	1	1	− 0.602	0.5	1*	1
Netcore, m10	2	1	1	1	− 0.536	0.5	0	0
Netcore, m11	2	1	1	1	− 0.646	0.5	0	0
Netcore, m12	2	1	1	1	− 0.603	0.5	0	0
DOMINO, all	53	81	7	0.059	− 0.353	0.509	0.485	12
DOMINO, m1	22	37	1	0.16	− 0.29	0.364	1*	3
DOMINO, m2	6	15	1	1	− 0.569	0.667	0.318	44
DOMINO, m3	6	8	1	0.533	− 0.392	0.667	0	0
DOMINO, m4	4	3	1	0.5	− 0.711	0.75	1*	4
DOMINO, m5	6	6	1	0.4	− 0.165	0.5	1*	3
DOMINO, m6	6	8	1	0.533	− 0.305	0.5	0.75	8
DOMINO, m7	3	2	1	0.667	− 0.474	0.667	0.4	5

Relevant module statistics for the GLUT4 data results. The modules are described by their method and module number, where “all” denotes all modules returned by a given method. The Proportion Significant column denotes the proportion of nodes in the module that have an ECI significantly different from zero. Pathway Count refers to the number of significant pathways returned by ORA. An asterisk (“*”) denotes the largest value in a given column

**Fig. 4:**
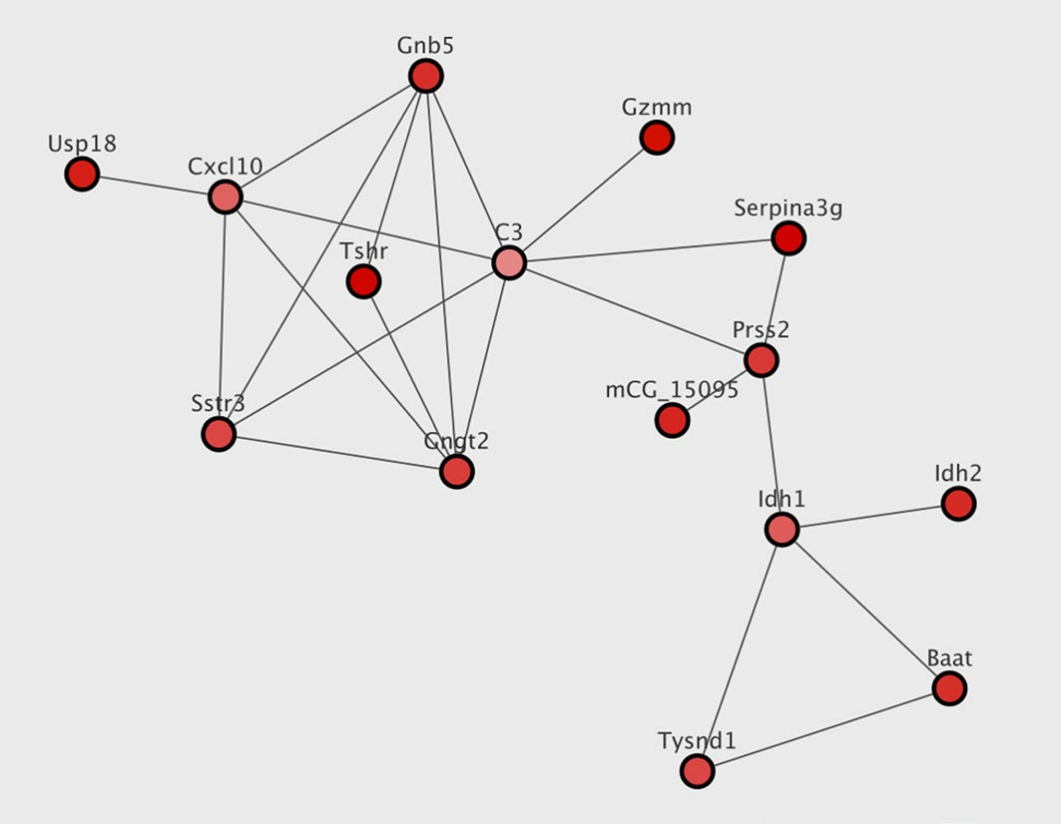
AMEND module for GLUT4 data. Darker shade of red signifies more extreme ECI

**Fig. 5:**
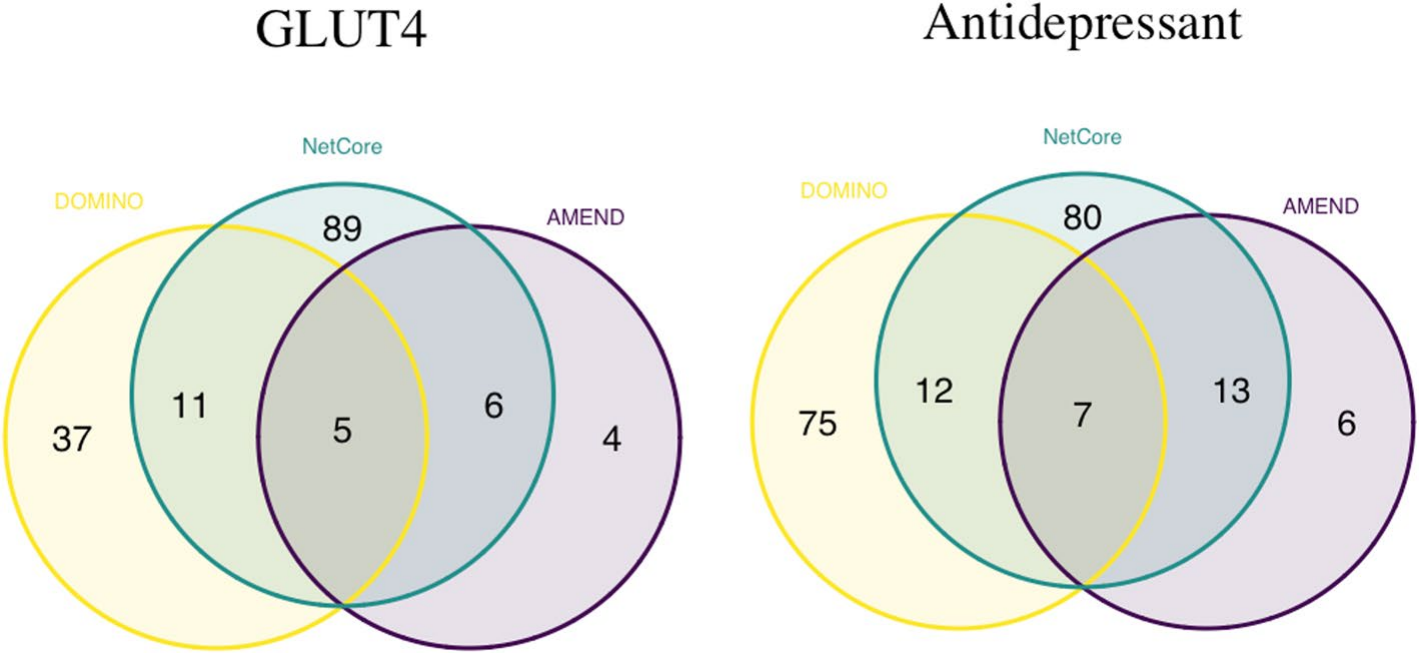
Node overlap between the final modules from AMEND, DOMINO, and NetCore

The AMEND module is visualized in Fig. 4 and has an EHR of 0.828, which corresponds to 24 EV pathways out of 29 total. Alterations to GLUT4 expression levels are associated with insulin sensitivity [21], and many of the returned pathways corroborate this finding. G protein activation and G alpha signaling are among the EV pathways. These signaling pathways play a key role in GLUT4 translocation to the plasma membrane, which is necessary for glucose transport into the cell [45]. There is also Glucagon-like Peptide-1 (GLP1) regulation of insulin secretion pathway. GLP1 has been shown to increase GLUT4 expression in adipose tissue [46]. Another interesting finding is the Aquaporin-mediated transport pathway. Aquaporins (AQPs) are emerging as important proteins in metabolic disorders, including insulin resistance and Type 2 Diabetes [47]. AQPs located in adipocytes have been shown to be co-regulated with GLUT4 when comparing insulin-resistant and lean human subjects [48]. DOMINO module 2 and NetCore module 1 share many of the same enriched pathways as those associated with the AMEND module. The three genes common to them all are TSHR, GNGT2, and GNB5. Figure 5 shows the node overlap between the methods. Interestingly, for both datasets there are no nodes common to only AMEND and DOMINO.

### Antidepressant data

The Antidepressant dataset involved the comparison of two antidepressant drugs, ketamine and imipramine, and their effects on gene expression in the prefrontal cortex of mice. For present purposes, it is of interest to identify genes that are similarly changed between the two treatment–control groups. This corresponds to genes with ECI values close to 1. As with the GLUT4 dataset, *n* was set to 15 for AMEND, resulting in a connected module of 26 nodes and 35 edges (Table 2). It has a median ECI of 0.706, being slightly less than that of NetCore module 3, although this module only consists of two nodes, which may be less biologically interesting than a module of larger size, because it defeats the purpose of looking at modules rather than individual genes. DOMINO module 1 has the largest proportion of significant ECI genes at 0.529, with the AMEND module and several others from NetCore and DOMINO having the second largest value of 0.5. Similar to the results from the GLUT4 dataset, AMEND retains both high-magnitude and significant ECI genes.

**Table 2:** Antidepressant data module statistics

Module	Nodes	Edges	Module count	Edge density	Median ECI	Proportion significant	EHR/mEHR	Pathway count
AMEND, n = 15	26	35	1	0.108	0.706	0.5	0.5	36
Netcore, all	112	207	5	0.033	0.691	0.366	0.359	95
Netcore, m1	104	203	1	0.038	0.69	0.385	0.353	119
Netcore, m2	2	1	1	1	0.478	0	0	0
Netcore, m3	2	1	1	1	0.708*	0.5	0	5
Netcore, m4	2	1	1	1	0.527	0	0	0
Netcore, m5	2	1	1	1	0.125	0	0	0
DOMINO, all	94	127	9	0.029	0.266	0.415	0.774	36
DOMINO, m1	17	32	1	0.235	0.538	0.529*	0.74	50
DOMINO, m2	20	34	1	0.179	0.059	0.4	0.932	59
DOMINO, m3	13	17	1	0.218	0.018	0.385	0.391	23
DOMINO, m4	10	11	1	0.244	0.166	0.4	1*	1
DOMINO, m5	6	6	1	0.4	0.43	0.5	1*	3
DOMINO, m6	7	6	1	0.286	0.392	0.429	0	0
DOMINO, m7	12	12	1	0.182	0.238	0.25	0	0
DOMINO, m8	4	3	1	0.5	0.423	0.5	1*	1
DOMINO, m9	5	4	1	0.4	0.095	0.4	1*	1

Relevant module statistics for the Antidepressant data results. The modules are described by their method and module number, where “all” denotes all modules returned by a given method. The Proportion Significant column denotes the proportion of nodes in the module that have an ECI significantly different from zero. Pathway Count refers to the number of significant pathways returned by ORA. An asterisk (“*”) denotes the largest value in a given column.

The EHR for AMEND is 0.5, corresponding to 18 EV pathways out of 36 total. Among these EV pathways are several associated with the Fibroblast Growth Factor Receptor (FGFR) signaling pathway. FGFR signaling is involved in the regulation of various neuronal processes, including proliferation and survival [49], and has been shown to be related to depression [49–51]. DOMINO module 2 had an mEHR of 0.932, consisting of 55 EV pathways, several of which are associated with GPCR signaling. Interestingly, GPCRs and FGFRs have been observed forming heterocomplexes in regulating the cell fate of neurons [49]. These two modules may be capturing related aspects of the biological processes impacted by the two antidepressants. Several of the EV pathways enriched for the AMEND module were also enriched for NetCore module 1, including the FGFR and ERBB2/ERBB4 signaling pathways.

Overall, the AMEND module gave the most extreme median ECI for the GLUT4 dataset, while coming in close second to NetCore module 3 for the Antidepressant dataset (median ECI of 0.706 for AMEND vs. 0.708 for NetCore). Also, AMEND returned the modules with the first and second largest proportions of significant ECI nodes for the GLUT4 and Antidepressant datasets, respectively. This shows that AMEND can outperform the benchmark algorithms in terms of capturing extreme ECI nodes.

### Consistency and sensitivity analysis

Consistency analysis aims to determine if AMEND returns similar modules obtained from independent datasets studying similar biological conditions. For the ECI modules, the maximum mean Jaccard Index was 0.053, for N = 10, whereas the maximum mean Nested Index was 0.333, for N = 10. All instances of non-zero overlap between pairs of modules were statistically significant at the 0.05 level. Pathway analysis also shows that many of the modules, after excluding the shared genes, were associated with various stages in the cell cycle (see Additional file 11).

The performance of AMEND may be unduly affected by changing parameter values. Therefore, a sensitivity analysis was done for the grid size of the restart parameter ($\alpha$) grid search and for the seed weight parameter *c*. There was little variation in median ECI, proportion of significant ECI nodes, mean core-clustering coefficient, or network score across a range of *c* values, suggesting that the algorithm is robust to changes in this parameter (see Additional files 3–6). AMEND was also run using a constant $\alpha$ of 0.2, 0.5, or 0.9, or grid sizes of 2, 4, 8, 16, and 18 on the GLUT4 and Antidepressant datasets. There wasn’t large variation but generally, performance increased with grid size. Interestingly, only a constant value of 0.5 had a significant negative effect. The constant values of 0.2 and 0.9 gave similar performances to the larger grid sizes (see Additional files 7–10).

## Discussion

In this study, we proposed a novel AMI method, AMEND, that incorporates two previously existing network analysis methods: RWR and *Heinz*, a heuristic solution to the MWCS problem. This is an iterative procedure that filters out genes at each step. The filtering rate is determined by an exponential decay schedule and allows flexibility in the size of the final module. The iterative nature of AMEND offers an advantage not present in other methods that perform the RWR procedure only once on the full PPI network [12, 13, 15, 52, 53]. PPI networks are static representations of protein interactions and thus will be noisy when viewed in the context of specific biological conditions. By performing RWR on smaller and more context-specific networks (as evidenced by an increasing $\overline{Z}$), the algorithm is better able to augment the biological signal present in the experimental data. AMEND also utilizes node coreness for its adjacency matrix normalization scheme in RWR, first introduced by the authors of NetCore, to attenuate degree bias inherent in PPI networks [13]. AMEND was developed for the ECI, which allows for the comparison of effect sizes between experiments. However, with slight modifications, AMEND can be generalizable to other feature-level summaries of the experimental data, such as log_2_ fold change. Other data types or different biological questions of interest may necessitate new seeding schemes for RWR. Regarding the PPI network, the intention behind setting a high threshold for the edge score was to limit false positives, i.e., spurious connections between genes. However, this does come with the increased risk of false negatives and may preclude some biologically relevant genes from being included in the final module.

Whereas many AMI methods return several disconnected modules of genes, each potentially representing different biological functions affected by the experimental treatments, AMEND returns a single, connected subnetwork. This can facilitate interpretation of findings and lead to new insights, since this connected subnetwork may include explicit interactions linking genes from different functional groups. For example, the AMEND module from the GLUT4 data contains two cliques of genes associated with different biological functions (see Fig. 4). The first clique consists of C3, CXCL10, GNB5, GNGT2, and SSTR3, and is associated with G alpha signaling events, while the second clique contains BAAT, IDH1, and TYSND1, and is associated with peroxisomal protein import. These two sets of genes are connected by PRSS2 and SERPINA3G. Further expertise and research would be required to determine if there is a plausible biological connection between these two functional groups of genes and what role PRSS2 and SERPINA3G might play in that connection.

The three algorithms were compared on the GLUT4 and Antidepressant datasets. Each dataset was associated with a specific PPI network that was derived by only retaining proteins that mapped to genes involved in the experiment. With respect to the GLUT4 dataset, NetCore returned the most modules at 12, with largely different sizes. There is one large module and several smaller modules (mostly of size 2). A similar observation is seen for NetCore with the Antidepressant dataset. For the GLUT4 dataset, both DOMINO and NetCore returned modules with mEHRs of 1. These were mostly small modules associated with 1–4 significantly enriched pathways each. AMEND had a relatively high mEHR of 0.828 while still capturing 29 enriched pathways, which were highly specific to G protein activation and signaling. 5 genes were common to the modules of all 3 algorithms: TSHR, SERPINA3G, PRSS2, GNB5, and GNGT2. TSHR, GNB5, and GNGT2 are associated with G proteins and G protein-coupled receptors, while SERPINA3G and PRSS2 are associated with serine proteases [54]. With respect to the Antidepressant dataset, AMEND returned a module with a relatively large median ECI and a high proportion of significant ECI genes. Its median ECI was only slightly exceeded by a NetCore module of size 2, which is not as biologically interesting as a larger module with an approximately equal median ECI. These results show that AMEND can identify subsets of genes that are connected, have large experimental values, and represent relevant and specific biological functions.

Additional analyses were carried out for the AMEND algorithm to determine its sensitivity to certain parameters and its consistency across independent datasets. The sensitivity analysis showed that results were fairly robust to changes in the seed weight parameter c, while performance tended to increase with an increasing grid size for the restart parameter grid search. In most cases, modules obtained from independent datasets did share common genes, albeit with limited overlap. Additionally, the non-zero Jaccard and Nested Indices measuring the extent of overlap were statistically significant, determined using a bootstrap approach. Given the differences in assay platform, time, location, and dosage between the four experiments, it is encouraging to find shared genes that are relevant biologically; EGFR, CDK6, and CDC20 play key functions in cell cycle progression and have been implicated in bladder cancer [55–57]. Not only were the shared genes highly relevant to bladder cancer, but the non-overlapping genes were also associated with relevant biological processes, such as cell cycle and DNA replication (see Additional file 11).

There are several limitations associated with this study. Comparisons of AMI methods and their results are rendered difficult by different factors. There is a lack of a gold standard for AMI methods, and the ground truth of the mechanisms underlying a given biological condition is usually unknown. Thus, the biological plausibility of results must be approximated through pathway analysis. Also, the algorithms included in this study return either a set of disconnected modules or a single, connected module. It is not entirely clear how these different types of results should be compared. There are also limitations with respect to the scope of this study. There is a plethora of AMI methods that have been developed on different data types using different techniques for filtering out nodes. However, we purposely restricted the benchmark methods to those that were developed for gene expression data and employ network diffusion, to facilitate fair comparisons. DOMINO and NetCore were included in this study for their high performance, their use of network diffusion, and their novelty, which includes the use of core normalization in RWR for NetCore and the development of the EHR for DOMINO.

The work presented in this study could be extended in several ways. As mentioned previously, it is generalizable to other data types, only requiring a modification to the RWR seed scheme. Also, the integration of several different omics data types could provide a more systematic description of the biological processes being studied. For example, transcriptomic and proteomic data are well suited to be integrated together with PPI networks. Other omics data may require different molecular interaction networks.

In summary, this study introduced AMEND, a novel AMI method that utilizes network diffusion in combination with the ECI to identify a connected subset of genes that are regulated in similar or opposing ways between two experimental conditions. It incorporates powerful network analysis techniques to filter out genes and was shown to outperform other AMI methods in terms of the median ECI and the proportion of significant ECI genes of the returned modules. AMEND is easily accessible as an R package.

## Supplementary Information

**Additional file 1**. Grid Search vs. PSO Comparison, GLUT4. PSO vs. grid search for starting filtering rate using GLUT4 dataset.

**Additional file 2**. Grid Search vs. PSO Comparison, Antidepressant. PSO vs. grid search for starting filtering rate using Antidepressant dataset.

**Additional file 3**. Seed weight sensitivity analysis: Median ECI. Sensitivity analysis on GLUT4 and Antidepressant data to determine how median ECI changes with seed weight.

**Additional file 4**. Seed weight sensitivity analysis: Proportion of significant ECI nodes. Sensitivity analysis on GLUT4 and Antidepressant data to determine how Proportion of significant ECI nodes changes with seed weight.

**Additional file 5**. Seed weight sensitivity analysis: Core-clustering coefficient. Sensitivity analysis on GLUT4 and Antidepressant data to determine how mean core-clustering coefficient changes with seed weight.

**Additional file 6**. Seed weight sensitivity analysis: Network score. Sensitivity analysis on GLUT4 and Antidepressant data to determine how network score changes with seed weight.

**Additional file 7**. Grid size sensitivity analysis: Median ECI. Sensitivity analysis on GLUT4 and Antidepressant data to determine how median ECI changes with restart parameter grid size. Left of the dashed line represents a constant restart value given in parentheses. Right of the dashed line represents grid size.

**Additional file 8**. Grid size sensitivity analysis: Proportion of significant ECI nodes. Sensitivity analysis on GLUT4 and Antidepressant data to determine how Proportion of significant ECI nodes changes with restart parameter grid size. Left of the dashed line represents a constant restart value given in parentheses. Right of the dashed line represents grid size.

**Additional file 9**. Grid size sensitivity analysis: Core-clustering coefficient. Sensitivity analysis on GLUT4 and Antidepressant data to determine how mean core-clustering coefficient changes with restart parameter grid size. Left of the dashed line represents a constant restart value given in parentheses. Right of the dashed line represents grid size.

**Additional file 10**. Grid size sensitivity analysis: Network score. Sensitivity analysis on GLUT4 and Antidepressant data to determine how network score changes with restart parameter grid size. Left of the dashed line represents a constant restart value given in parentheses. Right of the dashed line represents grid size.

**Additional file 11**. Consistency Analysis. Sheet 1: Results of consistency analysis, showing the Jaccard Index and the Nested Index for various comparison groups across different module sizes. Sheet 2: List of genes found in common between at least two modules from the BBN mouse data. Sheet 3: ORA was performed on all the modules, excluding the shared genes. This table shows the number of modules each pathway was associated with. The purpose was to assess the biological plausibility of the genes not shared between modules.

## Data Availability

The datasets supporting the findings of this study are available within the article.

## Abbreviations

PPI: Protein–protein interaction
ECI: Equivalent change index
AMI: Active module identification
RWR: Random walk with restart
MWCS: Maximum-weight connected subgraph
KO: Knockout
OX: Overexpression
DOI: Direction of interest
PCST: Prize-collecting Steiner tree
PSO: Particle swarm optimization
ORA: Overrepresentation analysis
EHR: Empirical-to-hypergeometric ratio
mEHR: Module-level empirical-to-hypergeometric ratio
EV: Empirically validated
