# Supplementary figures and images for: AMEND: active module identification using experimental data and network diffusion
Source: BMC Bioinformatics. 2023 Jul 6;24:277. doi: 10.1186/s12859-023-05376-z (PMC10324253; doi:10.1186/s12859-023-05376-z)

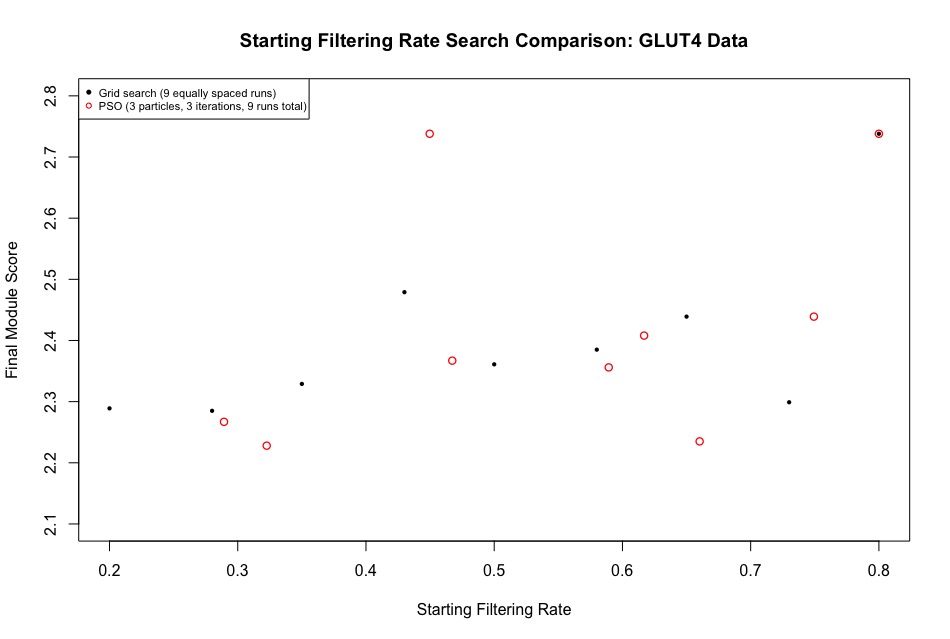

Supplement: Supplementary file 1 — Additional file 1. Grid Search vs. PSO Comparison, GLUT4. PSO vs. grid search for starting filtering rate using GLUT4 dataset. [file 12859_2023_5376_MOESM1_ESM.jpeg]

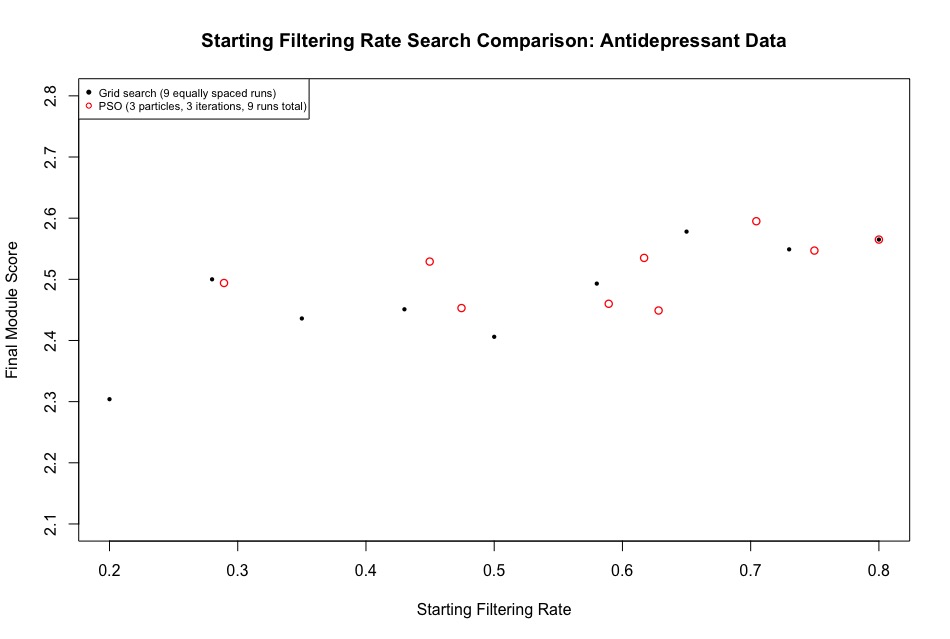

Supplement: Supplementary file 2 — Additional file 2. Grid Search vs. PSO Comparison, Antidepressant. PSO vs. grid search for starting filtering rate using Antidepressant dataset. [file 12859_2023_5376_MOESM2_ESM.jpeg]

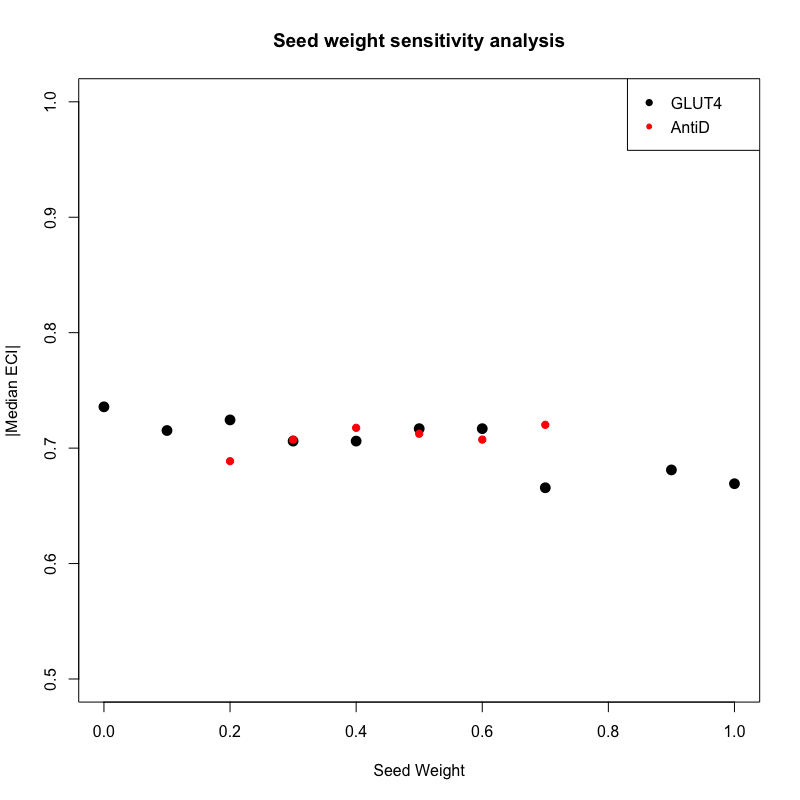

Supplement: Supplementary file 3 — Additional file 3. Seed weight sensitivity analysis: Median ECI. Sensitivity analysis on GLUT4 and Antidepressant data to determine how median ECI changes with seed weight. [file 12859_2023_5376_MOESM3_ESM.png]

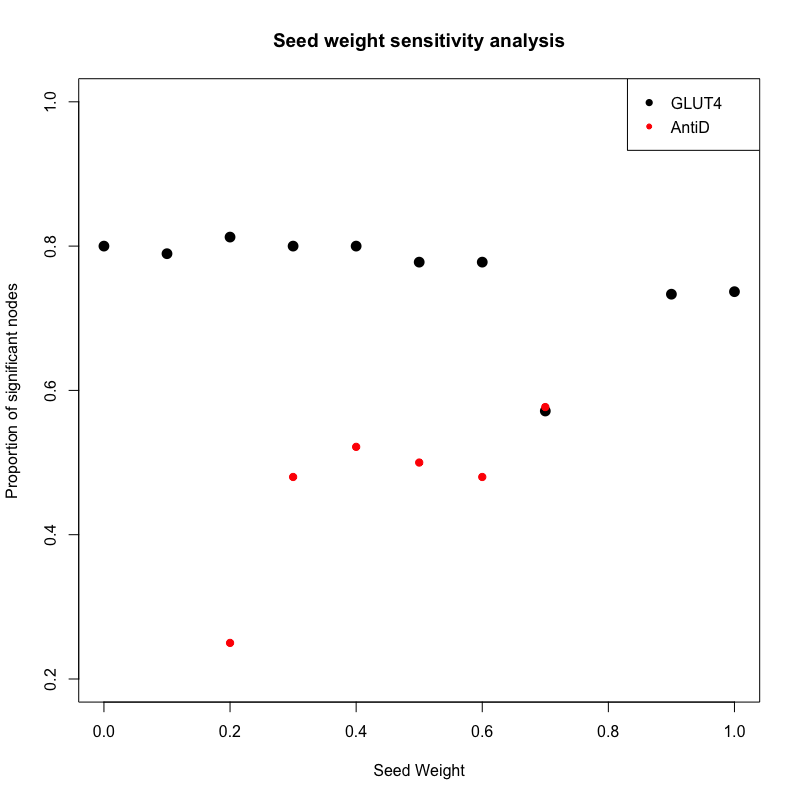

Supplement: Supplementary file 4 — Additional file 4. Seed weight sensitivity analysis: Proportion of significant ECI nodes. Sensitivity analysis on GLUT4 and Antidepressant data to determine how Proportion of significant ECI nodes changes with seed weight. [file 12859_2023_5376_MOESM4_ESM.png]

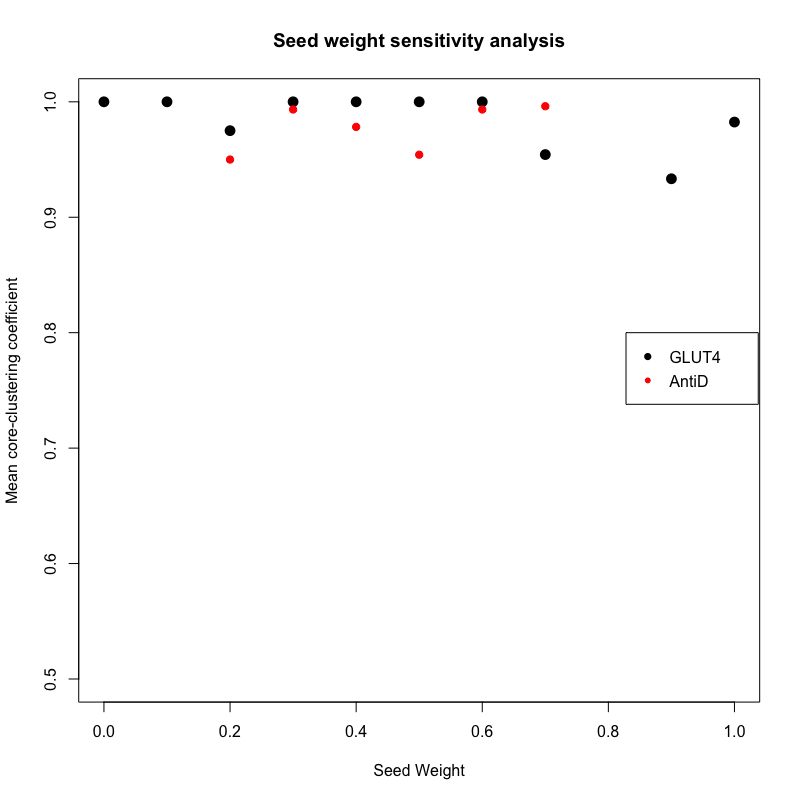

Supplement: Supplementary file 5 — Additional file 5. Seed weight sensitivity analysis: Core-clustering coefficient. Sensitivity analysis on GLUT4 and Antidepressant data to determine how mean core-clustering coefficient changes with seed weight. [file 12859_2023_5376_MOESM5_ESM.png]

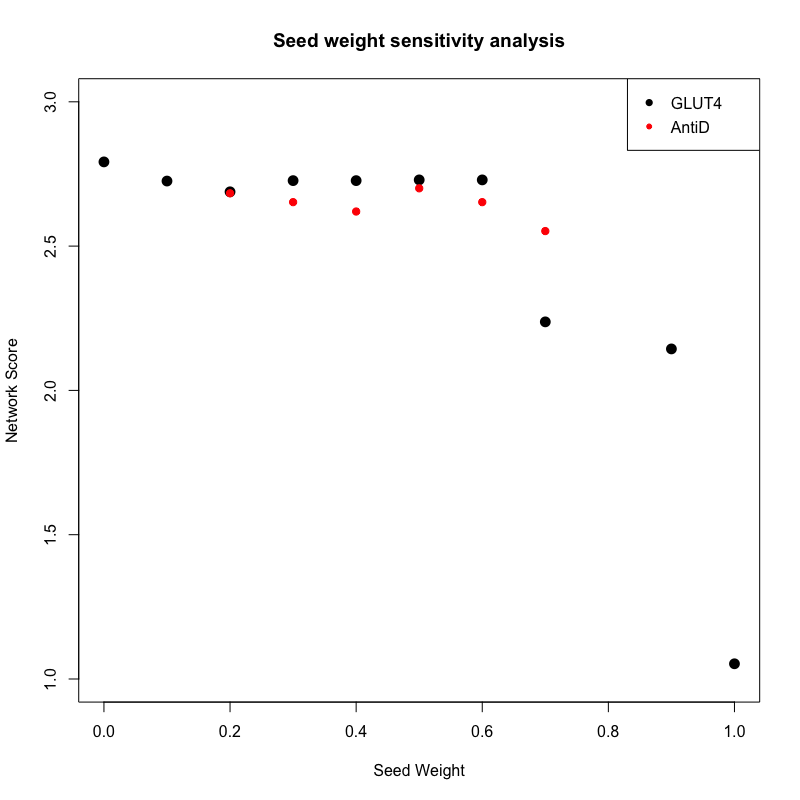

Supplement: Supplementary file 6 — Additional file 6. Seed weight sensitivity analysis: Network score. Sensitivity analysis on GLUT4 and Antidepressant data to determine how network score changes with seed weight. [file 12859_2023_5376_MOESM6_ESM.png]

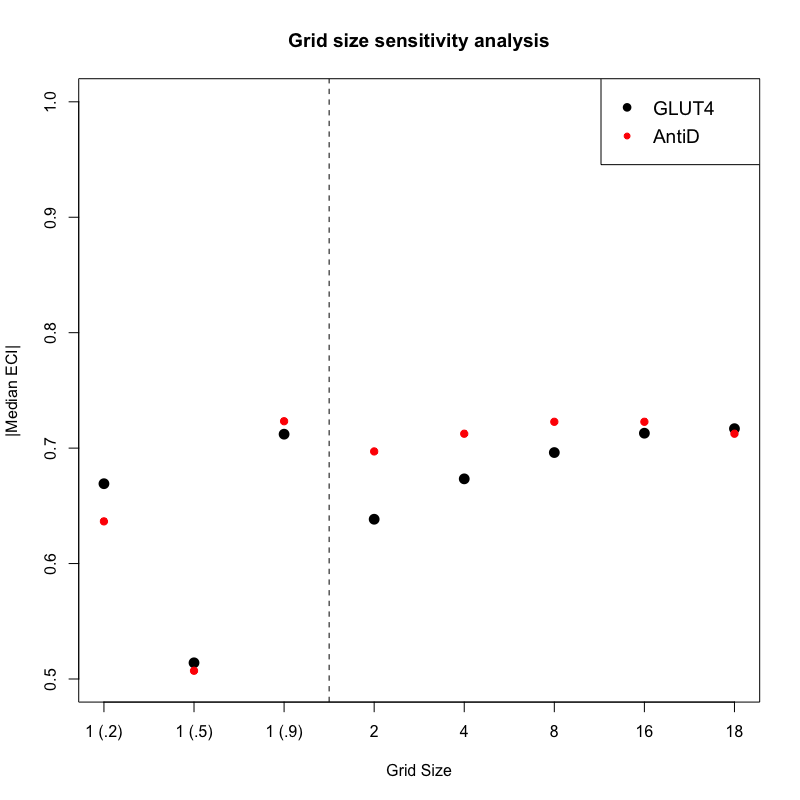

Supplement: Supplementary file 7 — Additional file 7. Grid size sensitivity analysis: Median ECI. Sensitivity analysis on GLUT4 and Antidepressant data to determine how median ECI changes with restart parameter grid size. Left of the dashed line represents a constant restart value given in parentheses. Right of the dashed line represents grid size. [file 12859_2023_5376_MOESM7_ESM.png]

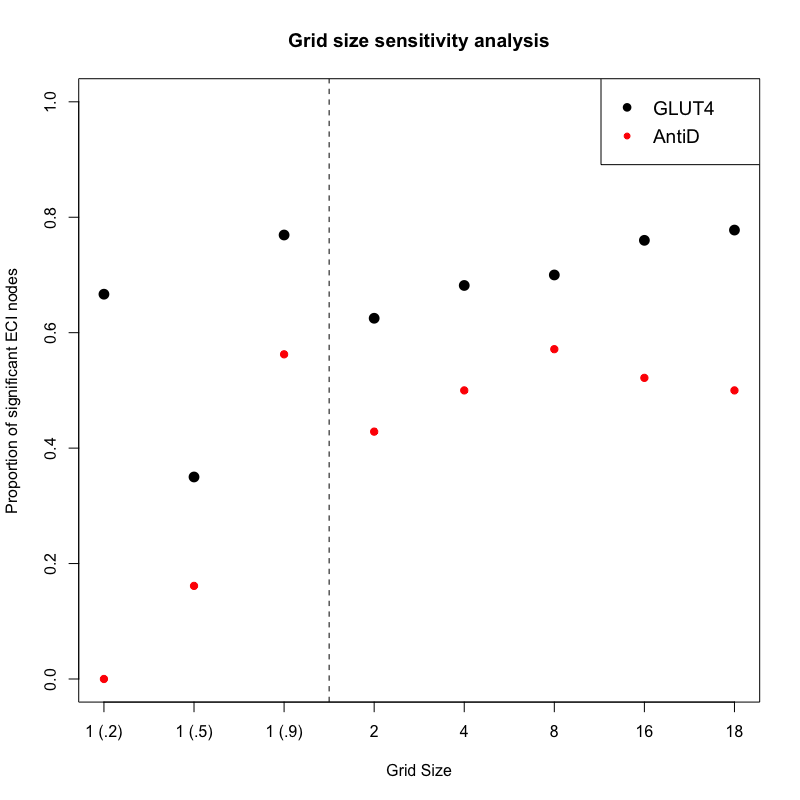

Supplement: Supplementary file 8 — Additional file 8. Grid size sensitivity analysis: Proportion of significant ECI nodes. Sensitivity analysis on GLUT4 and Antidepressant data to determine how Proportion of significant ECI nodes changes with restart parameter grid size. Left of the dashed line represents a constant restart value given in parentheses. Right of the dashed line represents grid size. [file 12859_2023_5376_MOESM8_ESM.png]

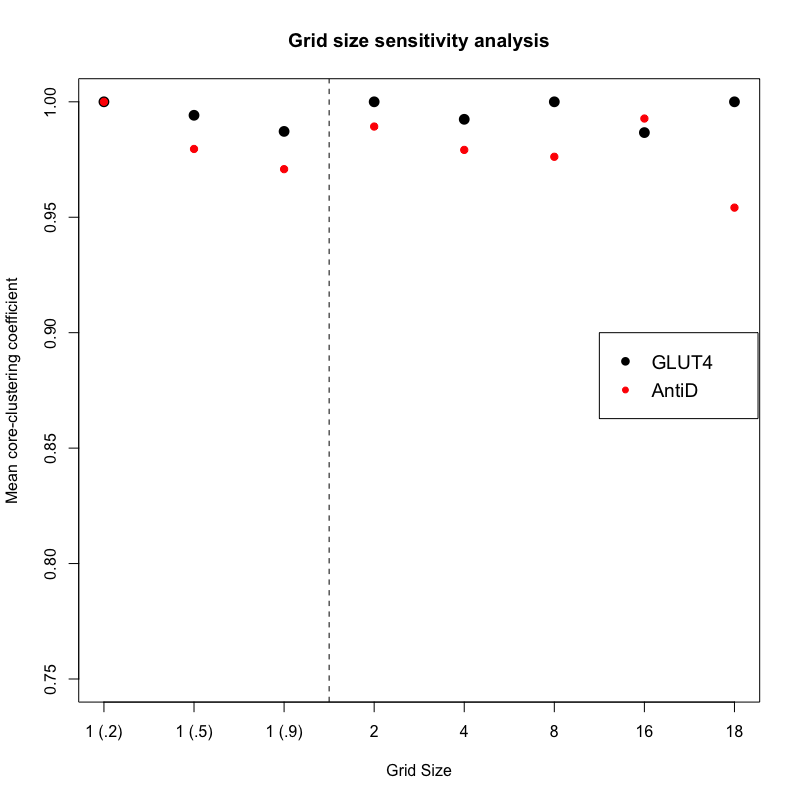

Supplement: Supplementary file 9 — Additional file 9. Grid size sensitivity analysis: Core-clustering coefficient. Sensitivity analysis on GLUT4 and Antidepressant data to determine how mean core-clustering coefficient changes with restart parameter grid size. Left of the dashed line represents a constant restart value given in parentheses. Right of the dashed line represents grid size. [file 12859_2023_5376_MOESM9_ESM.png]

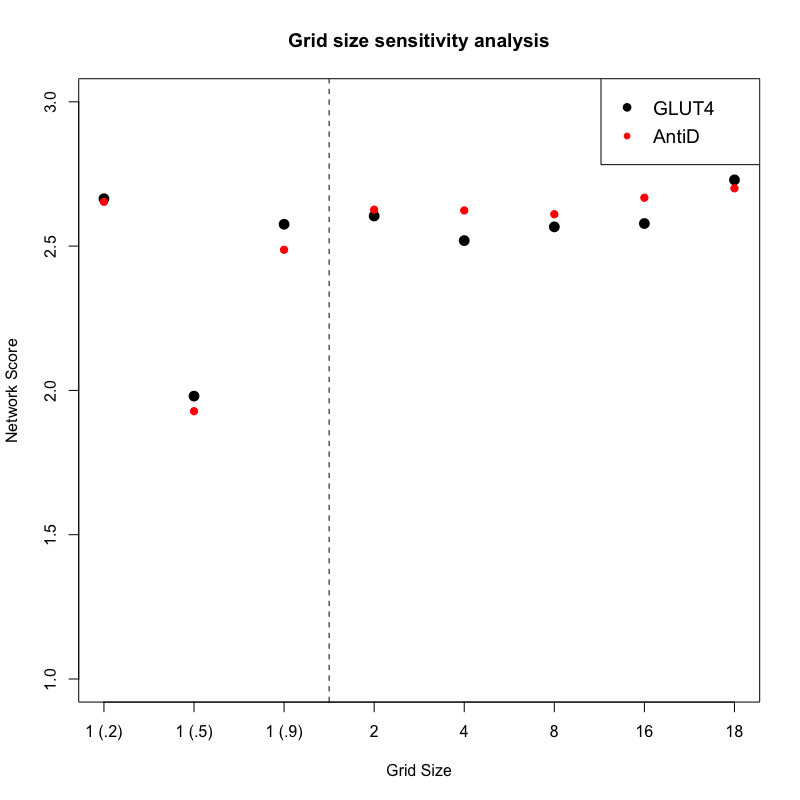

Supplement: Supplementary file 10 — Additional file 10. Grid size sensitivity analysis: Network score. Sensitivity analysis on GLUT4 and Antidepressant data to determine how network score changes with restart parameter grid size. Left of the dashed line represents a constant restart value given in parentheses. Right of the dashed line represents grid size. [file 12859_2023_5376_MOESM10_ESM.png]
